# Investigating the influence of breastfeeding on asthma in children under 12 years old in the UK Biobank

**DOI:** 10.3389/fimmu.2022.967101

**Published:** 2022-09-29

**Authors:** Wenyan Hou, Fengjun Guan, Lei Xia, Yue Xu, Shuiping Huang, Ping Zeng

**Affiliations:** ^1^ Department of Biostatistics, School of Public Health, Xuzhou Medical University, Xuzhou, China; ^2^ Department of Pediatrics, Affiliated Hospital of Xuzhou Medical University, Xuzhou, China; ^3^ Center for Medical Statistics and Data Analysis, Xuzhou Medical University, Xuzhou, China; ^4^ Key Laboratory of Human Genetics and Environmental Medicine, Xuzhou Medical University, Xuzhou, China; ^5^ Key Laboratory of Environment and Health, Xuzhou Medical University, Xuzhou, China; ^6^ Engineering Research Innovation Center of Biological Data Mining and Healthcare Transformation, Xuzhou Medical University, Xuzhou, China

**Keywords:** breastfeeding, children-onset asthma, sibling comparison analysis, UK Biobank, logistic regression

## Abstract

**Background:**

Childhood-onset asthma (COA) has become a major and growing problem worldwide and imposes a heavy socioeconomic burden on individuals and families; therefore, understanding the influence of early-life experiences such as breastfeeding on COA is of great importance for early prevention.

**Objectives:**

To investigate the impact of breastfeeding on asthma in children under 12 years of age and explore its role at two different stages of age in the UK Biobank cohort.

**Methods:**

A total of 7,157 COA cases and 158,253 controls were obtained, with information regarding breastfeeding, COA, and other important variables available through questionnaires. The relationship between breastfeeding and COA were examined with the logistic regression while adjusting for available covariates. In addition, a sibling analysis was performed on 398 pairs of siblings to explain unmeasured family factors, and a genetic risk score analysis was performed to control for genetic confounding impact. Finally, a power evaluation was conducted in the sibling data.

**Results:**

In the full cohort, it was identified that breastfeeding had a protective effect on COA (the adjusted odds ratio (OR)=0.875, 95% confidence intervals (CIs): 0.831~0.922; *P*=5.75×10^-7^). The impact was slightly pronounced in children aged 6-12 years (OR=0.852, 95%CIs: 0.794~0.914, *P=*7.41×10^-6^) compared to those aged under six years (OR=0.904, 95%CIs: 0.837~0.975, *P*=9.39×10^-3^), although such difference was not substantial (*P*=0.266). However, in the sibling cohort these protective effects were no longer significant largely due to inadequate samples as it was demonstrated that the power was only 23.8% for all children in the sibling cohort under our current setting. The protective effect of breastfeeding on COA was nearly unchanged after incorporating the genetic risk score into both the full and sibling cohorts.

**Conclusions:**

Our study offered supportive evidence for the protective effect of breastfeeding against asthma in children less than 12 years of age; however, sibling studies with larger samples were warranted to further validate the robustness our results against unmeasured family confounders. Our findings had the potential to encourage mothers to initiate and prolong breastfeeding.

## Introduction

Asthma, a reversible chronic airway inflammatory disease, is a major and growing problem worldwide, especially in developed countries. With population increase, economic growth and urbanization, the prevalence of asthma and allergic diseases among Chinese children has also elevated rapidly (1, 2), imposing a heavy socioeconomic burden on individuals and families. Available treatments can reduce morbidity of asthma, but is hard to alter the natural history of this disease (3). Therefore, identifying modifiable risk factors for asthma is a public health priority.

Particularly, how early-life experiences such as breastfeeding affect children-onset asthma (COA) has attracted much attention in the literature (4). As well-known, breast milk is the best source of nutrition for babies, and breastfeeding possesses many health benefits, including the prevention of gastroenteritis, atopic eczema (5) and respiratory infections (6) in the first year of life, and the reduction of childhood obesity risk (7, 8). Because of these advantages, the American Academy of Pediatrics and WHO strongly recommend exclusive breastfeeding for the first six months of life and encourage continued breastfeeding up to two years of age, adding other foods as needed.

There are several high-quality systematic reviews that have examined in detail the evidence on the impact of breastfeeding on COA. For instance, Dogaru et al. (4) demonstrated that breastfeeding could reduce the risk of asthma in both studies analyzing “asthma ever” and “recent asthma”, and that this protective effect was not influenced by study design or study quality or between studies in Western and non-Western countries. Lodge et al. (9) showed that there existed evidence that breastfeeding was protective against asthma for childhood of 5-18 years old; Güngör et al. (10) suggested that feeding human milk for short durations or not at all was associated with higher COA risk; and Xue et al. (11) found that the duration and exclusivity of breastfeeding were related to lower risk of asthma in children aged less than 7 years. However, there are also other studies which have found that breastfeeding appeared not to prevent asthma, delay its onset, or reduce its severity (12, 13) or concluded that breastfeeding was not likely to have substantial effect on the risk of developing asthma in the whole population (14). Differences in the conclusion of those studies are partly due to methodological issues relevant to sample size, study quality, or adjustment for confounders (15, 16). In particular, the association is largely influenced by genetic, environmental, and socioeconomic factors related to the pattern and duration of infant feeding and child health outcomes (17).

In the present study, we utilized the UK Biobank data to investigate the effect of breastfeeding on asthma in children under 12 years of age. It needs to first highlight that limiting the age of onset of asthma less than 12 years old is mainly because that asthma is the most common chronic non-communicable disease in pediatrics, affecting 5-10% of school-age children and adolescents (18), and that the protective effect of breastfeeding on asthma might be strongest in early childhood (19, 20). Previous studies detected the relationship between breastfeeding and asthma risk across distinct age groups from 2 months to 18 years, resulting in the likelihood that other exposures influencing this association increased with age (21).

Specifically, we analyzed the UK Biobank full cohort (7,157 COA cases and 158,253 controls) to investigate the influence of breastfeeding on asthma in children under 12 years of age and understand its role at two different stages of age in the UK Biobank cohort. We further sought to validate the discovered association using the UK Biobank sibling data (398 COA case siblings and 408 non-affected siblings) to control for the impact of family factors and incorporating a polygenic score of COA to adjust for the effect of genetic factors.

## Materials and methods

### Data sources

#### Full cohort in the UK Biobank

We first applied the UK Biobank full cohort to study the impact of breastfeeding on asthma among children under 12 years of age, which involved a total of 337,196 independent participants of white British ancestry (22). Following the definition used in a recent study that also employed the UK Biobank data (14), we chose COA cases as those individuals who had a self-report of physician-diagnosed asthma, with a question asked by “has a doctor ever told you that you have had any of the conditions below?”, in which “asthma” was one of the options listed. Note that, the large sample size of the UK Biobank cohort makes our analysis relatively robust against the exact diagnosis criteria of COA (14). Meanwhile, we ensured that each selected case had a corresponding age of asthma onset, leaving 37,573 participants with asthma and 9,125 asthma cases under the age of 12.

In order to maximize sample size to enhance power and guarantee the effectiveness of controls, we included all individuals without asthma as controls but filtered out those with asthma-related diseases regardless of whether their age of onset was less than 12 years (23), leaving 212,303 controls. These diseases included eczema/dermatitis (2,178 cases in total and 467 with onset age less than 12 years old), allergic rhinitis (18,402 cases in total and 4,351 with onset age less than 12 years old), allergic reactions (482 cases in total with no onset age), chronic obstructive pulmonary disease (COPD) (907 case in total but no cases with onset age less than 12 years old), emphysema/chronic bronchitis (2,342 cases in total and 211 with onset age less than 12 years old), bronchiectasis (649 cases in total and 66 with onset age less than 12 years old), sarcoidosis (287 cases in total and 2 with onset age less than 12 years old), and tuberculosis (372 cases in total and 178 with onset age less than 12 years old).

**Figure 1:**
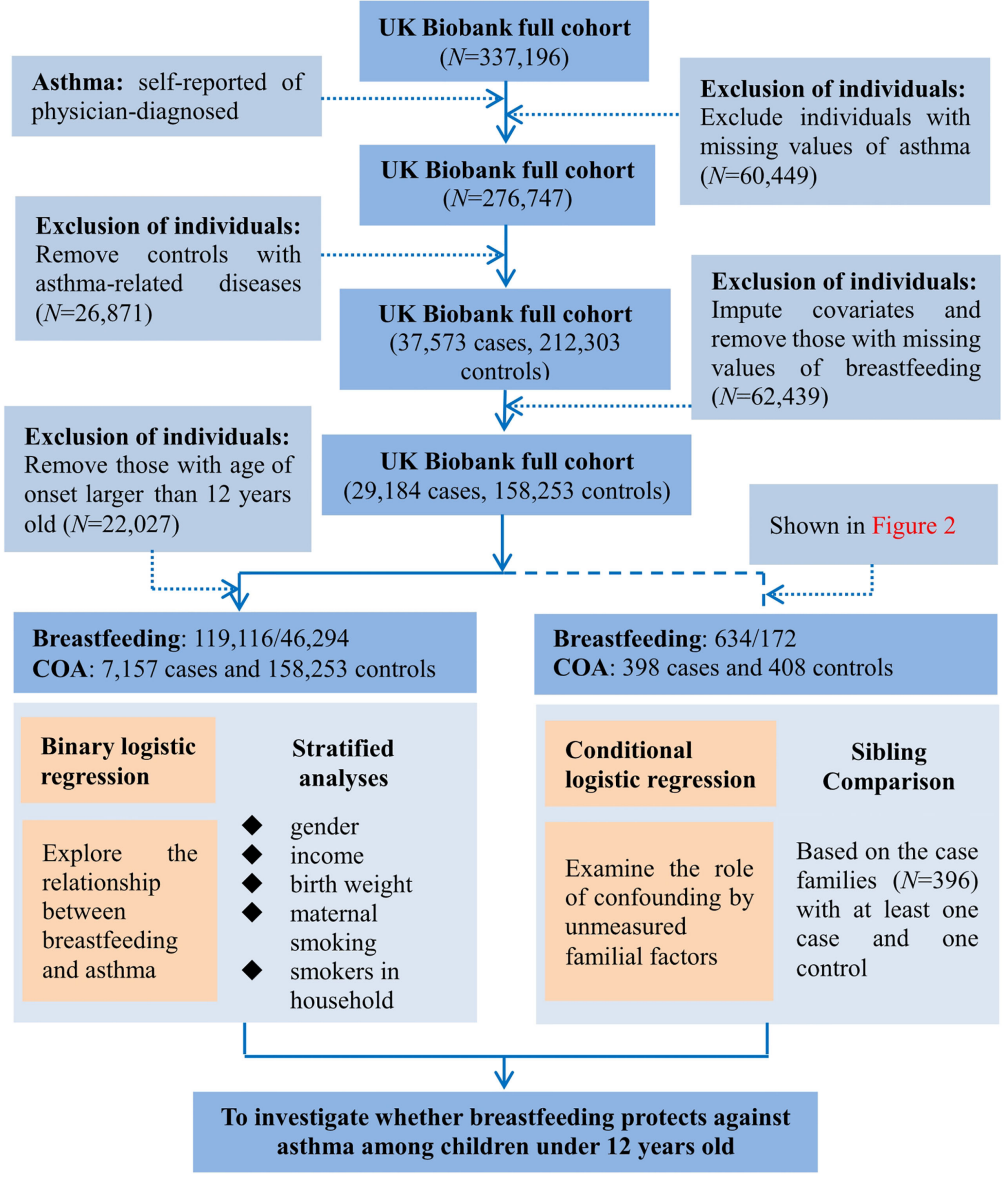
Flow diagram of data process and statistical analysis for the present study using the UK Biobank cohort.

In addition to breastfeeding (defined by the question: were you breastfed when you were a baby)? and COA defined above, we included birth weight, maternal smoking around birth, smoking/smokers in household, average total household income before tax, and gender as potential covariates (14, 24–26), but did not consider other important confounders such as family history of asthma, maternal gestational age, and maternal/paternal educational level because they were not available in the UK Biobank data (14). We note that there was a prior study which showed that the effect of breastfeeding might be not influenced by parental history of asthma (24). When covariates had missing values, we imputed continuous variables by the mean and categorical variables by the mode; but excluded participants with missing values for breastfeeding and asthma status. Afterwards, we reserved 7,157 COA cases and 158,253 controls in final analyses. The flow diagram of data processing is displayed in **Figure 1** and descriptive statistics of involved variables are shown in **Table 1** and **Table S1**.

**Table 1:** Descriptive statistics of variables in the UK Biobank full data after quality control used in the present work.

Variables	Cases	Controls	P value
Study population	7,157	158,253	
Breastfeeding (1/0)	5021/2136	114,095/44,158	3.47×10-4
Average total household income before tax (1/0)	4,741/2,416	91,839/66,414	7.69×10-43
Birth weight (kg) (mean ± sd)	3.36 ± 0.52	3.32 ± 0.53	1.23×10-7
Maternal smoking around birth (1/0)	1,922/5,235	41,801/116,452	0.408
Smoking/smokers in household (2/1/0)	88/575/6,494	1,574/12,871/143,808	0.358
Gender (1/0)	4,026/3,131	68,301/89,952	1.11×10-103

sd: standard deviation; breastfeeding was defined by asking “were you breastfed when you were a baby?” the answer was “yes” or “no”, coded as 1 or 0; maternal smoking around birth was defined by asking “did your mother smoke regularly around the time when you were born?” the answer was “yes” or “no”, coded as 1 or 0, and smoking/smokers in household was defined by asking “does anyone in your household smoke?” the answer was “yes, one household member smokes” (coded as 1), “yes, more than one household member smokes” (coded as 2), “no” (coded as 0), respectively, and was treated as a continuous variable; average total household income before tax was defined by asking “what is the average total income before tax received by your household?” The answer was “less than £18,000”, “£18,000 to £30,999”, “£31,000 to £51,999”, “£52,000 to £100,000”, and “greater than £100,000”, respectively; the five levels were classified as “less than” or “equal to or above” £31,000 and coded as 0 or 1, which was close to the UK median household income in 2009 (£27,530) (27). The gender was coded as 1 for boy or 0 for girl. The P value of the difference between these variables is shown in the last column.

#### Sibling cohort in the UK Biobank

**Figure 2:**
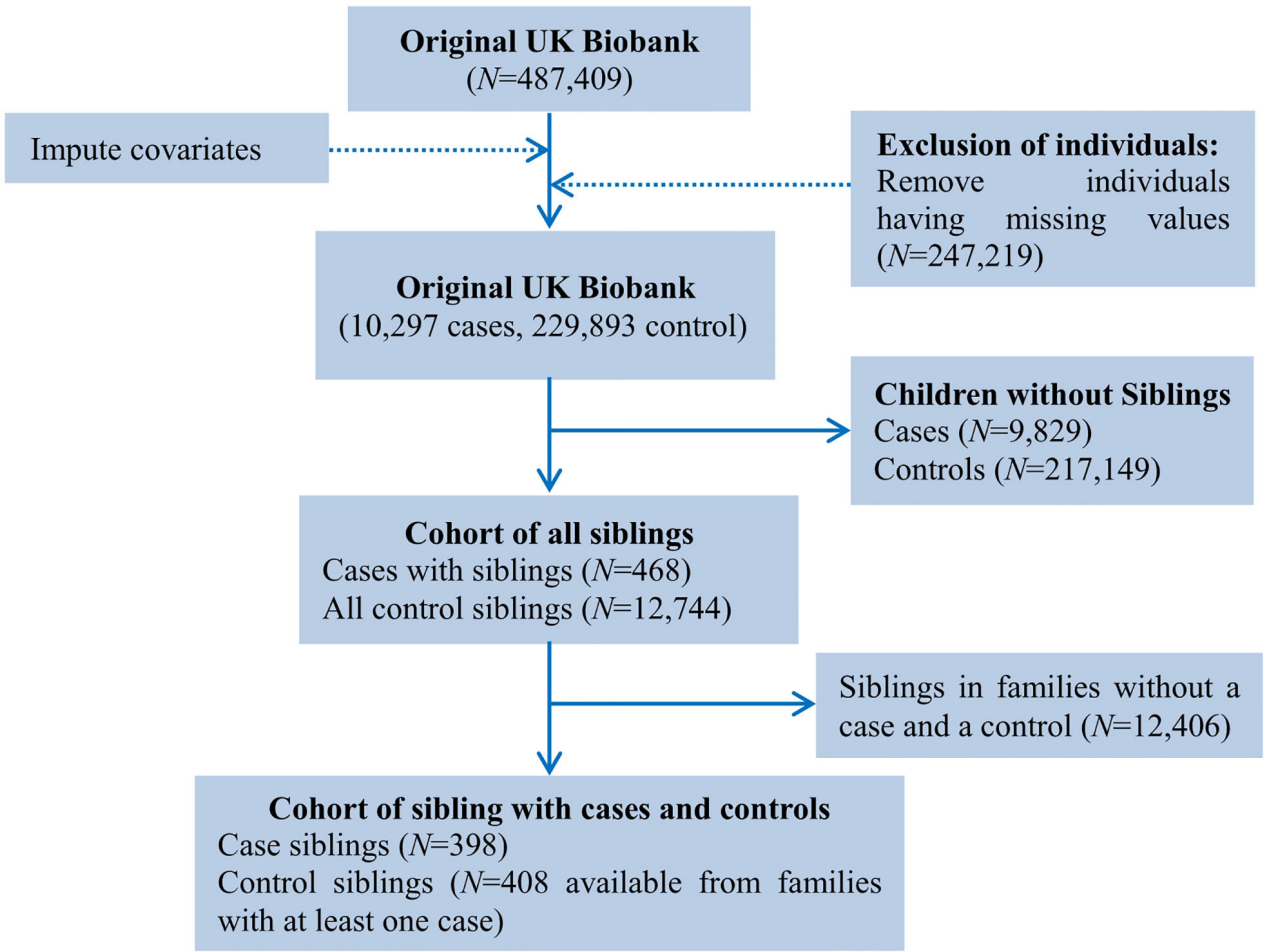
Flow chart of sample derivation for the study of breastfeeding and asthma in children under the age of 12 in the UK Biobank cohort.

In the full cohort, we were generally difficult to take the influence of family factors (e.g., maternal/paternal education) into account. Therefore, to adjust for the confounding effect of unmeasured familial factors, we implemented a sibling comparison analysis restricting to the group of cases with siblings (28, 29). To this aim, we identified genetically related individuals in the UK Biobank full cohort (*N*=487,409) *via* the KING software (30), and reported kinship coefficients for pairs of participants whom we inferred to be third-degree relatives or closer (31). Ultimately, a total of 147,731 UK Biobank participants (30.3%) were inferred to be related to at least one other person in the cohort, and formed a total of 107,162 related pairs, with 22,665 pairs of siblings. Again, we imputed missing values for covariates and removed siblings with missing values for breastfeeding and asthma, and finally reserved 396 families with COA cases. More specifically, we selected all cases (<12 years) from a specific family which had a case sibling (*n*=398) and at least one non-affected sibling of asthma cases (*n*=408). The flow diagram for sibling comparison study is illustrated in **Figure 2**.

### Statistical analysis

#### Binary logistic regression

First, we performed a binary logistic regression to study the influence of breastfeeding on COA. Based on the finding yielded from previous work (32), we further in terms of the age of onset classified these asthma cases into two groups including “0-5 years old” and “6-12 years old”, and performed the binary logistic regression in each group separately to evaluate whether breastfeeding had the similar effect on the two groups. Then, a stratification analysis was further conducted in terms of gender (boy/girl) (33), maternal smoking around birth (yes/no) (5), smoking/smokers in household (0, 1, or 2) (34), average total household income before tax (<£31,000 or ≥£31,000) (27) and birth weight (<2.5kg for low birth weight, 2.5-4.0kg for normal birth weight, or ≥4.0kg for high birth weight) (35, 36). Our classification of birth weight into these groups was based on pediatrics and existing studies (37).

#### Conditional logistic regression

Conditional logistic regression was applied to analyze the UK Biobank sibling data to adjust for unmeasured family factors (29, 38). Following previous literature which included the order of birth as a covariate (39, 40), besides those covariates described above we here additionally contained a binary variable for birth order in our analysis according to the age of sibling to control for such effect.

#### Genetic risk score analysis to adjust for genetic impact

Furthermore, as COA consistently exhibits strong genetic background (41, 42), we thus considered genetic risk score (GRS) of COA an approximation of genetics and performed a GRS analysis (27) in both the full and sibling data to assess the robustness of the observed association. Specifically, we selected single nucleotide polymorphisms (SNPs) associated with COA from (42), where 84 independent COA-associated SNPs were ultimately reserved (**Tables S2, S3**); the genome-wide significance criterion of 3.0×10^-8^ was used as suggested by (43). Then, the GRS was calculated *via* a weighted sum of the number of risk alleles for each participant (44, 45), and incorporated it into our analysis serving as another covariate.

#### Power evaluation in the sibling data

The small samples of the sibling cohort might lead to the concern of low power; therefore, we calculated the power through Lachin’s formula (46), in which the main parameters involved the desirable power for assessing whether breastfeeding was associated with the risk of COA, the odds ratio (OR), and the proportion of breastfeeding in the population (denoted by *p_b_*). We assessed the power with the estimated OR in our real-data analysis and the observed *p_b_* in the sibling cohort, and could obtain the corresponding sample size under varying expected power. The sample size calculated here represented a pair of case and control.

## Results

### Estimated effect size of breastfeeding on COA in the UK Biobank full cohort

**Figure 3:**
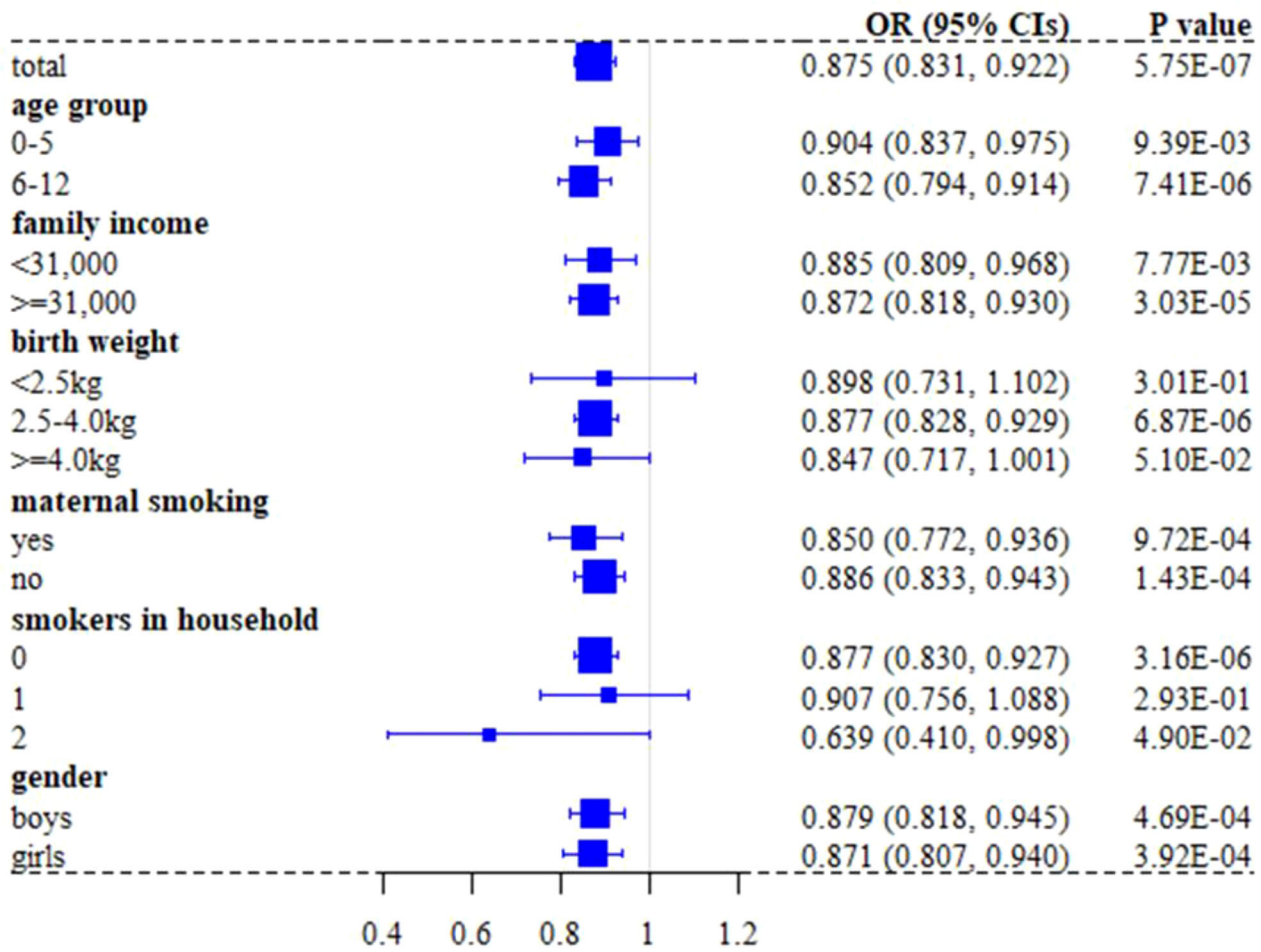
Forest plot of effect sizes of breastfeeding on asthma in children under 12 years old when stratified by covariates in the UK Biobank full cohort.

We found in the UK Biobank full cohort that breastfed children were generally less likely to develop asthma compared to those individuals who were not (the crude OR=0.910; 95% confidence intervals (CIs): 0.864~0.958, *P=*3.47×10^-4^). The effect slightly increased after considering available covariates (e.g., birth weight, maternal smoking around birth, smoking/smokers in household, average total household income before tax, and gender), with the adjusted OR=0.875 (95%CIs: 0.831~0.922, *P=*5.75×10^-7^) (**Table 2**). In addition, the CIs overlapped across the strata for each covariate in the stratification analysis (**Figure 3**), indicating the absence of interaction between breastfeeding and each covariate and further implying that the protective impact of breastfeeding on COA did not significantly differ in distinct levels of every covariate (**Tables S4, S5**). For example, the adjusted OR was 0.852 (95%Cls: 0.794~0.914, *P=*7.41×10^-6^) for children aged 6-12, while the adjusted OR was 0.904 (95%CIs: 0.837~0.975, *P*=9.39×10^-3^) for those under six years old; however, we did not find significant difference between the two estimated effects (*P*=0.266). The results of stratification analysis for asthma under six years old and aged 6-12 are shown in **Figures S1, S2**. In contrast to the protective effect of breastfeeding, all considered covariates elevated the risk of developing COA.

**Table 2:** Estimated effect sizes and relationships between breastfeeding and COA in the UK Biobank full cohort.

Variables	X—>Y		X—>Y1		X—>Y2	
	OR (95%CIs)	P value	OR (95%CIs)	P value	OR (95%CIs)	P value
Breastfeeding	0.875 (0.831, 0.922)	<0.001	0.904 (0.837, 0.975)	0.009	0.852 (0.794, 0.914)	<0.001
Average total household income before tax	1.396 (1.328, 1.468)	<0.001	1.246 (1.159, 1.339)	<0.001	1.543 (1.441, 1.653)	<0.001
Birth weight	1.069 (1.022, 1.118)	0.004	1.060 (0.992, 1.132)	0.083	1.077 (1.014, 1.145)	0.016
Maternal smoking around birth	1.009 (0.956, 1.065)	0.746	1.082 (1.002, 1.170)	0.045	0.950 (0.883, 1.022)	0.169
Smoking/smokers in household	1.034 (0.964, 1.109)	0.348	1.055 (0.954, 1.167)	0.299	1.016 (0.924, 1.117)	0.744
Gender	1.680 (1.601, 1.763)	<0.001	1.350 (1.258, 1.447)	<0.001	2.028 (1.899, 2.166)	<0.001

X, breastfeeding; Y, asthma in children under 12 years old; Y1, asthma in children under six years old; Y2, asthma in children between 6-12 years old.

### Relationship between breastfeeding and COA in the UK Biobank sibling cohort

The estimated relationships between breastfeeding and COA in the sibling data are summarized in **Table 3**. It was observed that the magnitude and direction of effect estimates were analogous to those obtained in the full cohort, but the estimated effects were much less stable (i.e., with larger standard error) due to the relatively small number of discordant sibling pairs; therefore, the 95% CLs became wider. Specifically, the OR of breastfeeding was 0.806 (95%CIs 0.489~1.327) for asthma in children under 12 years old in the sibling cohort, and the ORs were 0.771 (95%CIs: 0.395~1.504) for asthma in children under six years old and 0.810 (95%CIs: 0.376~1.744) for asthma in children between 6-12 years old.

**Table 3:** Estimated effect sizes and relationships between breastfeeding and COA in the UK Biobank sibling cohort.

Variables	X—>Y		X—>Y1		X—>Y2	
	OR (95%CIs)	P value	OR (95%CIs)	P value	OR (95%CIs)	P value
Breastfeeding	0.806 (0.489, 1.327)	0.396	0.771 (0.395, 1.504)	0.446	0.810 (0.376, 1.744)	0.590
Average total household income before tax	0.901 (0.649, 1.250)	0.531	0.843 (0.536, 1.327)	0.461	0.943 (0.577, 1.541)	0.814
Birth weight	1.086 (0.788, 1.495)	0.615	1.065 (0.690, 1.642)	0.778	1.098 (0.677, 1.781)	0.705
Maternal smoking around birth	1.142 (0.680, 1.919)	0.615	1.262 (0.586, 2.717)	0.552	1.068 (0.523, 2.182)	0.857
Smoking/smokers in household	1.392 (0.926, 2.091)	0.112	1.232 (0.722, 2.101)	0.444	1.632 (0.857, 3.107)	0.136
Gender	1.681 (1.238, 2.283)	<0.001	1.761 (1.143, 2.712)	0.010	1.649 (1.057, 2.571)	0.027
Birth order	1.113 (0.912, 1.359)	0.291	1.156 (0.873, 1.531)	0.312	1.062 (0.795, 1.419)	0.683

X, breastfeeding; Y, asthma in children under 12 years old; Y1, asthma in children under six years old; Y2, asthma in children between 6-12 years old. Birth order was determined based on the age of sibling pairs. In the sibling cohort, there was one family with two cases and one control, and the two cases belonged to in the “0-5” and “6-12” age groups, respectively; thus, when classified by the age of onset, this family was divided into two families, resulting in the total number of controls (i.e., 409) one more than the original number of controls (i.e., 408).

### Influence of GRS on the estimated effect of breastfeeding on COA

The effect of breastfeeding on COA was almost unchanged after including GRS as an additional covariate for explaining the impact of genetic factors (**Tables S6, S7**). For instance, the ORs of breastfeeding for asthma were 0.876 (95%CIs: 0.831~0.923) for all childhood under 12 years old, 0.905 (95%CIs: 0.838~0.977) for childhood under six years old, and 0.853 (95%CIs: 0.795~0.915) for childhood aged between 6-12 years old in the full cohort, respectively; the corresponding ORs in the sibling cohort were 0.808 (95%CIs: 0.490~1.333), 0.771 (95%CIs: 0.395~1.505), and 0.822 (95%CIs: 0.380~1.776). The effects of GRS on COA were all significant in the full cohort (*P*=6.95×10^-7^, 0.011, and 8.13×10^-6^ for all childhood under 12 years old, childhood under six years old, or childhood aged between 6-12 years old, respectively); however, the effects of GRS on COA were non-significant in the sibling cohort (*P*=0.403, 0.446, and 0.617, respectively), largely due to inadequate samples of the sibling cohort (see below).

### Expected power and sample size in the sibling cohort

**Figure 4:**
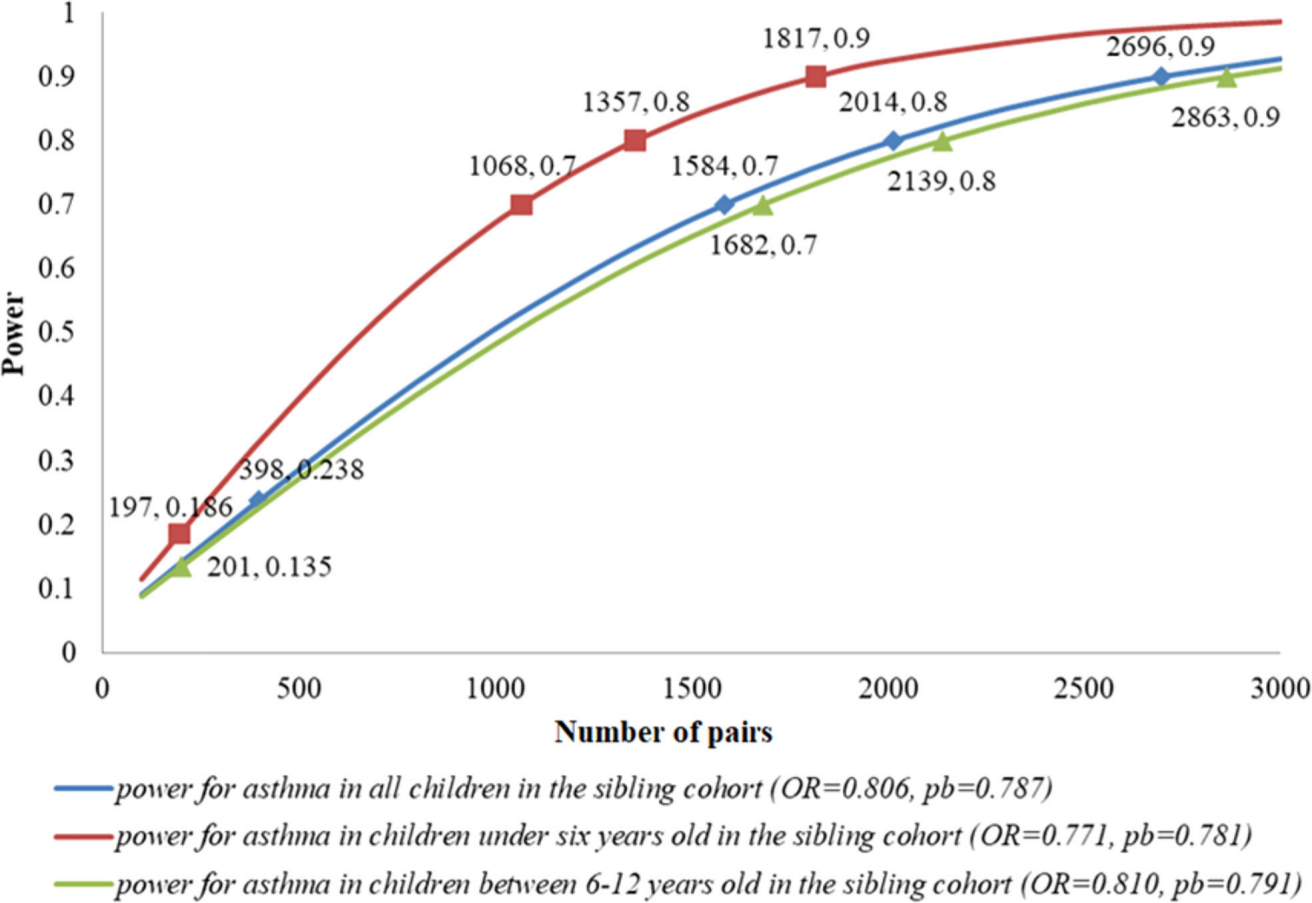
Expected power and sample size in the sibling cohort. These sample sizes were calculated under the estimated OR in the real-data analysis and the observed population of breastfeeding (pb) in the sibling cohort. For each pair of numbers labeled in the plot, the first one denoted the number of pairs, and the second one denoted the power under this setting.

As shown in **Figure 4**, under the current setting of *N*=398, OR=0.806 and *p_b_* =0.787, the power was only 23.8% for all children in the sibling cohort. If we expected the power to increase up to 70%, we needed 1,584 pairs of siblings. For children under six years old in the sibling cohort, within the current setting of *N*=197, OR=0.771 and *p_b_* =0.781, the power was equal to 18.6%; 70% power required 1,068 pairs of siblings. For children between 6-12 years old in the sibling cohort, under the current setting of *N*=201, OR=0.810 and *p_b_* =0.791, the power was only 13.5%; 70% power needed 1,682 pairs of siblings.

## Discussion

### Summary of our results

In the present study, we identified the protective effect of breastfeeding on asthma in children under 12 years old in the UK Biobank full cohort (22). In the sibling cohort study, the protective effect of breastfeeding on COA persisted; however, none of these effects was statistically significant. At least three explanations should be considered. First, the full cohort associations did at least in part result from familial confounding; second, fewer sibling pairs participated in the analysis lead to low power; third, non-differential measurement error of the exposure (i.e., random misclassification of breastfeeding) was present. Here, in terms of our power assessment, the reduced power in the sibling cohort was more possibly responsible for the non-significance. After taking the influence of genetic factors into account by adding a genetic risk score, we found that our estimated effect sizes were nearly unchanged, indicating that genetic factors of asthma did not likely confound the identified association.

### Comparison with previous studies

Our study was in line with and complementary to multiple existing studies (20, 47, 48), including a prior study performed on UK children born in 2002, which demonstrated eliminating “not breastfeeding” risk factor could prevent asthma (49), and a recent study carried out in Puerto Rican children, which showed breastfed for up to 6 months resulted in 30% lower risk of asthma than those who were not breastfed for children aged 6-14 years (50). Although we only analyzed breastfeeding or not, and not consider duration of breastfeeding, a latest population-based study (51) showed that longer breastfeeding duration was inversely associated with COA, which was in contrast the conclusion that duration of breastfeeding was not relevant to asthma at age 7 years (52). Therefore, the influence of breastfeeding duration on COA needs further investigation.

Among many prior studies, Ek et al (14) is the most analogous to our work, where the authors also utilized the UK Biobank data but failed to discover a beneficial impact of breastfeeding on asthma. The primary difference between our work and the study of (14) is that we focused only on COA, whereas Ek et al analyzed all asthma cases that could be available from the full UK Biobank cohort. It is well known that there exists considerable discrepancy between childhood-onset and adult-onset asthma (AOA) in environmental and genetic causes (42, 53–57). Specifically, it is believed that perinatal factors, atopy, viral respiratory-tract infections, and the microbiome often play a more critical role in the development of COA (58–61), while obesity, smoking, and other environmental and occupational exposures are more related to AOA. In addition, the two types of asthma show much different genetic foundations, with COA (heritability=25.6%) being more heritable compared to AOA (heritability=10.6%) (42).

These differences could certainly produce distinct consequence regarding the influence of breastfeeding on asthma. Indeed, we could further identify that breastfeeding showed a protective effect on asthma for asthma patients whose onset age was less than 40 (OR=0.904, 95%CIs 0.837~0.975 for 0-5 group, OR=0.852, 95%CIs 0.794~0.914 for 6-12 group, OR=0.803, 95%CIs 0.729~0.885 for 13-18 group, and OR=734 95%CIs 0.703~0.766 for 19-40 group), but displayed a deleterious impact on asthma for those with onset age larger than 40 (OR=1.017, 95%CIs 0.970~1.067 for 41-60 group, and OR=1.408, 95%CIs 1.236~1.603 for >60 group). The above finding suggests that the protective effect of breastfeeding on asthma cannot persist into adulthood and that the effect of breastfeeding on asthma is considerably heterogeneous in various age-onset groups. As a result, combining to analyze COA and AOA in the full cohort could cancel out the significant influence of breastfeeding on asthma in each group. This also indicates the reasonableness and validness of our study which investigated the relationship between breastfeeding and asthma only in one sub-group population of the UK Biobank cohort.

### Advantages and limitations of this work

Compared to previous work, our study possesses three major advantages. First, the datasets we analyzed were obtained from the UK Biobank with a much larger sample size than that of existing studies; therefore, the results were less biased by sampling error. Second, we conducted a sibling comparison design that explained the influence of family factors (28, 29). Third, we implemented a GRS analysis that took genetic factors into account (62). These design strategies guaranteed the validity of our results which were less likely confounded by unmeasured confounding factors.

Of course, the present study also has some limitations. First, there likely exists the misclassification of breastfeeding and COA. For example, information on breastfeeding and asthma in the UK Biobank was obtained from questionnaires and may be subject to recall bias as indicated by other retrospective work which also utilized the UK Biobank data under distinct application context (63). Specifically, the used UK Biobank data on breastfeeding and COA was collected between 2006 and 2010 with all participants older than 40, which resulted in a major concern that the further back in time the data was collected when breastfeeding occurred, the greater the recall bias may be. Thus, we could not completely remove the probability of recall bias which might drive the observed association between breastfeeding and COA (64).

Second, breastfeeding was simply categorized as yes/no, without considering duration and feeding methods (e.g., exclusive, or mixed breastfeeding). There were considerable variations in breastfeeding duration between studies (50, 65, 66), making comparisons across studies difficult and also not allowing us to explore the dose-response relationship between breastfeeding duration and COA. In our study, asthma was also incapable of distinguishing between mild and severe as well as between atopic and non-atopic (12).

Third, it remained a slight concern that the risk of reverse causality because early symptoms of allergic disease might already be present during breastfeeding and thus encouraged mothers to continue breastfeeding (67), which could dilute the protective effect of breastfeeding on asthma or even create the spurious consequence that breastfeeding increased asthma risk (14). Finally, due to data availability of the UK Biobank, we did not have some other important covariates such as family history of asthma, maternal gestational age, maternal or paternal educational level, children BMI and life styles; therefore, the present work cannot fully remove the influence of potential confounders.

### Public health implications of our findings

WHO has issued a recommendation that mothers should exclusively breastfeed their children for at least six months; However, it is reported that 81% of women in the UK do breastfeed at birth of babies, but fewer than half continue beyond 6 weeks (68). According to the UK Department of Health, the breastfeeding rate for babies aged 6-8 weeks in the UK in 2020-2021 was only 47.6%; according to the “Investigation Report on Influencing Factors of Breastfeeding in China” released by the China Development Research Foundation in February 2019, the exclusive breastfeeding rate of Chinese infants within 6 months was only 29%, which was much lower than the average of 43% worldwide and the average of 37% in low/middle-income countries. Therefore, our results had important implications for health and social policy for reducing COA, including increased breastfeeding education during pregnancy in health care facilities and paid maternity leave to promote and extend breastfeeding across all developed and developing countries. Particularly, the breastfeeding education program could be effective for sustaining breastfeeding in new mothers (69). The breastfeeding rate is influenced by individuals, medical institutions, and social structures (70). However, evaluating breastfeeding rate is not easy; for example, when breastfeeding in public, women are challenged by shared concerns around unwanted attention, coping with an awkward audience and unsuitable environments; women want to feel comfortable when breastfeeding in a public space (71).

In conclusion, our study offered supportive evidence for the protective effect of breastfeeding against asthma in children less than 12 years of age; however, sibling studies with larger samples were warranted to further validate the robustness our results against unmeasured family confounders. Our findings had the potential to encourage mothers to initiate and prolong breastfeeding.

## Data availability statement

Publicly available datasets were analyzed in this study. This data can be found here: https://www.ukbiobank.ac.uk/.

## Ethics statement

The UK Biobank had approval from the North West Multi-Centre Research Ethics Committee (MREC) as a Research Tissue Bank (RTB) approval. All participants provided written informed consent before enrolment in the study, which was conducted in accordance with the Declaration of Helsinki. This approval means that researchers do not require separate ethical clearance and can operate under the RTB approval.

## Author contributions

PZ conceived the idea for the study. PZ obtained the data. PZ, FG and WH cleared up the datasets and performed the data analyses. PZ, SH, YX, LX, FG and WH interpreted the results of the data analyses. PZ and WH wrote the manuscript with the participation of other authors. All authors contributed to the article and approved the submitted version.

## Funding

The research of PZ was supported in part by the National Natural Science Foundation of China (82173630 and 81402765), the Youth Foundation of Humanity and Social Science funded by Ministry of Education of China (18YJC910002), the Natural Science Foundation of Jiangsu Province of China (BK20181472), the China Postdoctoral Science Foundation (2018M630607 and 2019T120465), the Qinglan Project of Jiangsu Province for Middle and Young Academic Leaders, the Six-Talent Peaks Project in Jiangsu Province of China (WSN-087), the Training Project for Youth Teams of Science and Technology Innovation at Xuzhou Medical University (TD202008), the Postdoctoral Science Foundation of Xuzhou Medical University, and the Statistical Science Research Project from National Bureau of Statistics of China (2014LY112).

## Acknowledgments

This study was also based on the UK Biobank resource under application number 88159. The UK Biobank was established by the Wellcome Trust medical charity, Medical Research Council, Department of Health, Scottish Government, and the Northwest Regional Development Agency. It has also had funding from the Welsh Assembly Government, British Heart Foundation and Diabetes UK. The data analyses in the present study were carried out with the high-performance computing cluster that was supported by the special central finance project of local universities for Xuzhou Medical University. We thank the Editor and the Reviewers for their thorough and useful comments which substantially improved our manuscript.

## Conflict of interest

The authors declare that the research was conducted in the absence of any commercial and financial relationships that could be construed as a potential conflict of interest.

## Publisher’s note

All claims expressed in this article are solely those of the authors and do not necessarily represent those of their affiliated organizations, or those of the publisher, the editors and the reviewers. Any product that may be evaluated in this article, or claim that may be made by its manufacturer, is not guaranteed or endorsed by the publisher.
